# Sonographic Cortical Development and Anomalies in the Fetus: A Systematic Review and Meta-Analysis

**DOI:** 10.3390/biomedicines12071397

**Published:** 2024-06-24

**Authors:** Ilenia Mappa, Daniele Di Mascio, Luigi Carbone, Jia Li Angela Lu, Sara Sorrenti, Chiara Patelli, Alice D’Amico, Barbara Matarrelli, Giulia Andrea Giuliani, Daniele Neola, Raffaella Di Girolamo, Laura Sarno, Asma Khalil, Giuseppe Rizzo, Giuseppe Maria Maruotti, Francesco D’Antonio

**Affiliations:** 1 Department of Obstetrics and Gynecology, Fondazione Policlinico Tor Vergata, University of Rome Tor Vergata, 00133 Rome, Italychiarapatelli18@gmail.com (C.P.); giuseppe.rizzo@uniroma1.it (G.R.); 2 Department of Maternal and Child Health and Urological Sciences, Sapienza University of Rome, 00161 Roma, Italy; 3 Gynecology and Obstetrics Unit, Department of Neuroscience, Reproductive Sciences and Dentistry, University of Naples Federico II, 80131 Naples, Italy; 4 Center for Fetal Care and High-Risk Pregnancy, Department of Obstetrics and Gynecology, University of Chieti, 66100 Chieti, Italy; 5 Gynecology and Obstetrics Unit, Department of Public Health, University of Naples Federico II, 80131 Naples, Italygm.mar@tiscali.it (G.M.M.); 6 Fetal Medicine Unit, St George’s University Hospital, London SW17 0QT, UK; 7 Vascular Biology Research Centre, Molecular and Clinical Sciences Research Institute, St George’s University of London, London SW17 0RE, UK; 8 Twins Trust Centre for Research and Clinical Excellence, St George’s University Hospital, St George’s University of London, London SW17 0RE, UK

**Keywords:** ultrasound, cortical anomalies, lissencephaly, Sylvian fissure, neurosonography, systematic review

## Abstract

The aim of this systematic review is to report the normal cortical development of different fetal cerebral fissures on ultrasound, describe associated anomalies in fetuses with cortical malformations, and evaluate the quality of published charts of cortical fissures. The inclusion criteria were studies reporting development, anomalies, and reference charts of fetal cortical structures on ultrasound. The outcomes observed were the timing of the appearance of different cortical fissures according to different gestational age windows, associated central nervous system (CNS) and extra-CNS anomalies detected at ultrasound in fetuses with cortical malformation, and rate of fetuses with isolated anomaly. Furthermore, we performed a critical evaluation of the published reference charts for cortical development on ultrasound. Random-effect meta-analyses of proportions were used to combine the data. Twenty-seven studies (6875 fetuses) were included. Sylvian fissure was visualized on ultrasound in 97.69% (95% CI 92.0–100) of cases at 18–19, 98.17% (95% CI 94.8–99.8) at 20–21, 98.94% (95% CI 97.0–99.9) at 22–23, and in all cases from 24 weeks of gestation. Parieto-occipital fissure was visualized in 81.56% (95% CI 48.4–99.3) of cases at 18–19, 96.59% (95% CI 83.2–99.8) at 20–21, 96.85% (95% CI 88.8–100) at 22–23, and in all cases from 24 weeks of gestation, while the corresponding figures for calcarine fissure were 37.27% (95% CI 0.5–89.6), 80.42% (95% CI 50.2–98.2), 89.18% (95% CI 74.0–98.2), and 96.02% (95% CI 96.9–100). Malformations of cortical development were diagnosed as an isolated finding at ultrasound in 6.21% (95% CI 2.9–10.9) of cases, while they were associated with additional CNS anomalies in 93.79% (95% CI 89.1–97.2) of cases. These findings highlight the need for large studies specifically looking at the timing of the appearance of the different brain sulci. Standardized algorithms for prenatal assessment of fetuses at high risk of malformations of cortical development are also warranted.

## 1. Introduction

Advances in prenatal imaging techniques have led to a significant increase in the detection rate of fetal anomalies [1,2,3,4,5,6,7,8,9]. Central nervous system (CNS) anomalies are among the most common structural malformations detected in prenatal ultrasound. Most of the anomalies potentially identifiable before birth are commonly suspected in the standard axial view of fetal brain, which is the only imaging slice recommended for the ultrasound screening of fetal brain anomalies [2]. Conversely, the International Society of Ultrasound in Obstetrics and Gynecology (ISUOG) recommends that a multiplanar assessment of the fetal brain, the so-called neurosonography, using different axial, coronal, and sagittal views of the brain, be performed in case an anomaly is suspected during the screening assessment of the fetus [1]. Malformations of cortical development are a group of rare disorders commonly presenting with severe neurological symptoms, including developmental delays, cerebral palsy, and seizures [10,11]. The clinical phenotype of these anomalies is variable and mainly dependent upon the type, extent, and severity of the malformation and the involved genetic pathways of brain development. These anomalies are rarely present in isolation and are commonly diagnosed in the setting of other brain malformations, such as ventriculomegaly or midline or posterior fossa anomalies, and represent the main determinant of neurological outcome when associated with isolated CNS anomalies. Despite its importance, the assessment of fetuses at high risk of being affected by cortical anomalies is challenging. Published guidelines do not specifically state how to approach fetuses at high risk of cortical anomalies [1,2,12]. Furthermore, the development of cortical fissures on ultrasound has not been largely reported yet. Most studies are retrospective and include a very small number of cases, thus making it difficult to extrapolate an objective figure from the normal cortical development. Finally, only a few growth charts on cortical development have been published, and there is no study reporting their methodological quality. In this setting, we designed a systematic review and meta-analysis of the published literature to report the normal cortical development of the different cerebral fissures on ultrasound, describe the associated anomalies in fetuses with cortical malformations, and evaluate the quality of the published chats of cortical fissures.

## 2. Materials and Methods

### 2.1. Data Sources

This review was performed according to an a priori-designed protocol and recommended for systematic reviews and meta-analyses [13,14]. The Medline and Embase databases were searched electronically on 1 August 2023, utilizing combinations of the relevant medical subject heading (MeSH) terms, keywords, and word variants for “fetal cortex”, “cortical development”, and “lissencephaly” in line with current recommendations and reported as per PRISMA 2020 guidelines [15]. The search and selection criteria were restricted to the English language. Reference lists of relevant articles and reviews were hand-searched for additional reports. We registered our review on PROSPERO (registration number: CRD42023461602).

### 2.2. Main Outcome Measures

The inclusion criteria were studies reporting the development, anomalies, and reference charts of fetal cortical structures on ultrasound. The outcomes observed were:

- Timing of appearance of the different cortical fissures according to different gestational age windows (18–19, 20–21, 22–23, and >24 weeks);
- Associated CNS and extra-CNS anomalies detected at ultrasound in fetuses with cortical malformations and the rate of fetuses with an isolated anomaly;
- Critical evaluation of the published reference charts for cortical development using ultrasound.

For the assessment of the associated anomalies detected on ultrasound in fetuses with cortical malformations, we considered the following conditions: ventriculomegaly, midline, posterior fossa, and multiple anomalies. In order to report the quality of the published reference charts on fetal cortical fissures, we adopted the methodology proposed by Ioannu et al. [16] and adapted it to the brain charts by Corroenne et al. [17], consisting of the evaluation of the study design, statistical analysis, and reporting methods.

### 2.3. Eligibility Criteria

Only studies reporting the development, outcome, and reference charts of fetal cortical structures on ultrasound were considered eligible for inclusion in the present systematic review. Studies published before 2000 were also excluded, as we considered that advances in prenatal imaging techniques make them less relevant. Only full-text articles were considered eligible for inclusion; case reports, conference abstracts, and case series with fewer than 5 cases were excluded in order to avoid publication bias.

Quality assessment of the included studies reporting the rate of associated anomalies in fetuses with cortical anomalies and in those studies reporting the timing at appearance of the different cortical fissures was performed using the Newcastle–Ottawa Scale (NOS) [18]. According to the NOS, each study is judged from three broad perspectives: the selection of the study groups; the comparability of the groups; and the ascertainment of the outcome of interest. Assessment of the selection of a study includes the evaluation of the representativeness of the exposed cohort, selection of the non-exposed cohort, ascertainment of exposure, and demonstration that the outcome of interest was not present at the start of the study. Assessment of the comparability of the study includes the evaluation of the comparability of cohorts based on the design or analysis (i.e., whether the study controls for only the most important factor or also for any additional factor). Finally, the ascertainment of the outcome of interest includes the evaluation of the type of the assessment of the outcome of interest, length, and adequacy of follow-up. According to the NOS, a study can be awarded a maximum of one star for each numbered item within the Selection and Outcome categories. A maximum of two stars can be given for Comparability.

### 2.4. Data Collection and Analysis

Two authors (AL and CP) reviewed all abstracts independently. Agreement regarding potential relevance was reached by consensus. Full-text copies of those papers were obtained, and the same two reviewers independently extracted relevant data regarding study characteristics and pregnancy outcomes. Inconsistencies were discussed by the reviewers, and consensus was reached by discussion with a third author (FDA). If more than one study was published for the same cohort with identical endpoints, the report containing the most comprehensive information on the population was included to avoid overlapping populations. The overall quality score of the published charts on fetal cortical development was calculated for each study as the percentage of criteria classified as low risk of bias out of the total number of quality criteria.

We employed random-effect meta-analyses to combine data and reported their results as pooled proportions with their 95% confidence intervals (CIs) [19,20,21].

Between-study heterogeneity was explored using the I^2^ statistic, which represents the percentage of between-study variation that is due to heterogeneity rather than chance. A value of 0% indicates no observed heterogeneity, whereas I^2^ values of ≥50% indicate a substantial level of heterogeneity. All analyses were performed using StatsDirect Statistical Software 2.8.0 (StatsDirect Ltd. Cambridge, UK).

Funnel plots displaying the outcome rate from individual studies versus their precision (1/standard error) were carried out with an exploratory aim. Tests for funnel plot asymmetry were not used when the total number of publications included for each outcome was less than ten. In this case, the power of the tests is too low to distinguish chance from real asymmetry.

## 3. Results

### 3.1. General Characteristics of the Studies

**Figure 1:**
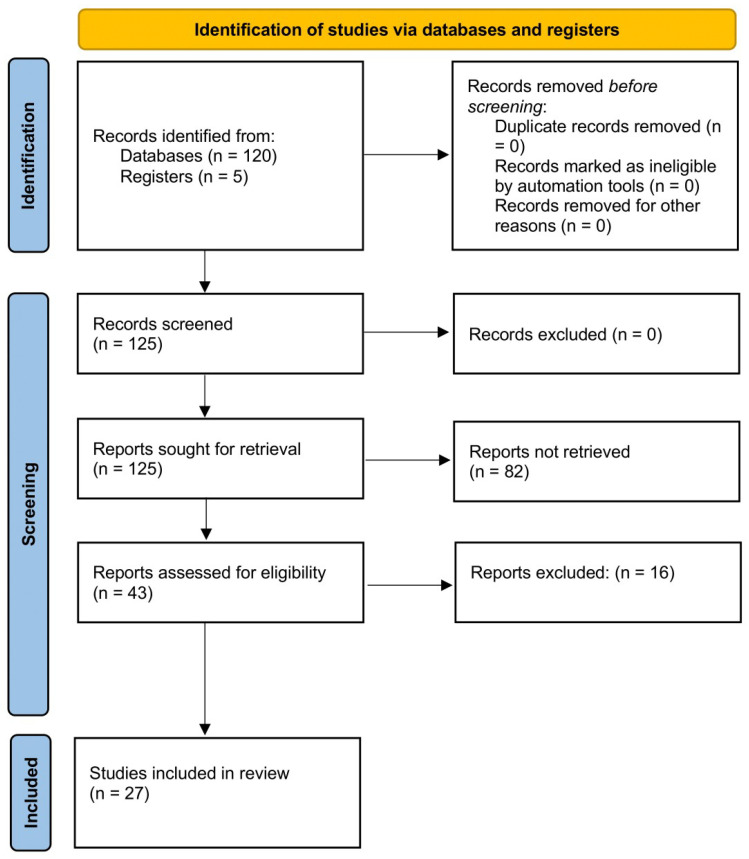
Systematic review flowchart.

Of a total of 125 articles that were identified, 43 were assessed with respect to their eligibility for inclusion, and 27 studies were included in the systematic review (Table 1, Figure 1, and Supplementary Table S1) [22,23,24,25,26,27,28,29,30,31,32,33,34,35,36,37,38,39,40,41,42,43,44,45,46,47,48]. These studies included 6875 fetuses undergoing neurosonography (after removing the studies including overlapped cases). Six studies (689 fetuses) reported the timing of the appearance of different cortical fissures on ultrasound, eight (145 fetuses) showed the rate of CNS and extra-CNS anomalies associated with cortical malformation, seven reported the refence ranges for different cerebral sulci (2636 fetuses), and six (3505 fetuses) studies reported the cortical development using 2D or 3D ultrasound. The results of the quality assessment of the included studies on fetal cortical development and anomalies using the NOS tool are presented in Table 2. The included studies showed an overall good score regarding the selection and comparability of the study groups and for ascertainment of the outcome of interest. Their major limitations were the retrospective design, small sample size, heterogeneity in the outcomes observed, and the inclusion of cases referred to detailed ultrasound assessment for other CNS and extra-CNS conditions other than the suspicion of cortical anomalies. No study mentioned differences according to fetal sex.

### 3.2. Synthesis of the Results

#### 3.2.1. Cortical Development and Associated Anomalies

Six studies (589 fetuses) explored the development of cortical structures on ultrasound, and a pooled data synthesis could only be performed for three cortical fissures (Sylvian, parieto-occipital, and calcarine fissure). A Sylvian fissure was visualized on ultrasound in 97.69% (95% CI 92.0–100) of cases at 18–19 weeks, 98.17% (95% CI 94.8–99.8) at 20–21 weeks, 98.94% (95% CI 97.0–99.9) at 22–23 weeks, and in all cases from 24 weeks of gestation. A parieto-occipital fissure was visualized in 81.56% (95% CI 48.4–99.3) of cases at 18–19 weeks, 96.59% (95% CI 83.2–99.8) at 20–21 weeks, 96.85% (95% CI 88.8–100) at 22–23 weeks, and in all cases from 24 weeks of gestation, while the corresponding figures for calcarine fissures were 37.27% (95% CI 0.5–89.6), 80.42% (95% CI 50.2–98.2), 89.18% (95% CI 74.0–98.2), and 96.02% (95% CI 84.7–100), respectively (Table 3). Malformations of cortical development were diagnosed as an isolated finding at ultrasound in 6.21% (95% CI 2.9–10.9) of cases, while they were associated with additional central nervous system (CNS) anomalies in 93.79% (95% CI 89.1–97.2) of cases. Regarding the different CNS anomalies associated with the occurrence of malformations of cortical development, isolated ventriculomegaly was reported in 6.20% (95% CI 0.7–16.7), midline anomalies were observed in 10.17% (95% CI 1.8–24.2), posterior fossa anomalies were reported in 2.24% (95% CI 0.4–5.4), and multiple anomalies were observed in 82.63% (95% CI 65.6–94.7) of fetuses undergoing neurosonography (Table 4).

#### 3.2.2. Evaluation of Published Charts of Cortical Development

Seven studies (2636 fetuses) reported the growth charts of different cortical fissures on ultrasound and were evaluated adopting the methodology proposed by Ioannu et al. [16] and adapted to brain charts, consisting of the evaluation of the study design, statistical methods, and reporting methods (Table 5). Regarding the study design, a high risk of bias was reported in 14.3% (1/7) of studies in the inclusion/exclusion criteria, 28.6% (2/7) in the sample selection, and in 57.1% (4/7) in sample size calculation and gestational age at inclusion. For the statistical methods, we reported a high risk of bias in 57.1% (4/7) studies in the number of measurements taken for each variable, assessment of increasing variability of data with gestation, assessment of the goodness-of-fit of the model, and in the methods used to estimate gestational age-specific intervals for the measurements. Finally, regarding the reported methods, we observed a high risk of bias in 57.1% (4/7 studies) in terms of the characteristics of the study population, in 42.9% (3/7) in terms of the description of the number approached/enrolled and in the reporting of regression equations, and in 71.4% (5/7) in the reporting of the mean and standard deviation of each measurement.

## 4. Discussion

The findings from this systematic review showed that there is still limited evidence on fetal cortical development in healthy fetuses. Sylvian and parieto-occipital fissures were visualized in about all cases at the time of the routine anomaly scan, while calcarine fissures were only detected in half of the included cases. The large majority of cortical anomalies presented in the setting of other CNS malformations, mainly ventriculomegaly or midline anomalies, were only detected in about 6% of cases presented in isolation. Finally, most of the published charts were affected by a high risk of bias in either the study design, statistical analysis, and reporting methods. This is, to the best of our knowledge, the first systematic review assessing cortical anomalies, their development, and the quality of the published charts. A thorough literature search, an assessment of either cortical development or anomalies, and a critical evaluation of the reported charts using a validated tool to assess growth charts reflect the main strengths of this study. The small number of included studies and even smaller number of cases presenting with isolated malformations of cortical development, heterogeneity in gestational age at assessment in studies reporting the anomalies, and lack of blinded assessment in those reporting the normal cortical development represents the main weaknesses of these studies. The lack of a standardized protocol to approach malformations of cortical development among the included studies represents another major limitation of this study. Most of the cases presenting with malformations of cortical development were referred for fetal neurosonography due to the presence of CNS or extra-CNS anomalies. Studies reporting cortical development were affected by the lack of inter- and intra-observer variability in estimating the presence of a given cortical fissure, the paucity of cerebral sulci assessed, and the lack of stratification of the analysis according to the type of ultrasound assessment adopted (trans-abdominal vs. trans-vaginal).

Prenatal identification of malformations of cortical development is challenging, mostly in terms of knowledge of the timing of their appearance throughout pregnancy. The International Society of Ultrasound of Ultrasound in Obstetrics and Gynecology suggested that parasagittal planes of fetal brains should be obtained, especially in the third trimester, in order to assess the normal development of the gyri of the cortex and the Sylvian fissure. Despite this, there is no specific mention on how to assess the normal cortical development in fetuses referred for neurosonography and which imaging signs should be used to diagnose a cortical anomaly [1,2]. Most of the published studies included a very small number of cases, mainly presenting with other major CNS or extra-CNS anomalies, thus making the identification of a cortical anomaly easier. The assessment of fetuses with suspected cortical anomalies implies an objective knowledge of the normal fetal cortical development, imaging signs suggesting an anomaly, and a quantitative evaluation of the brain sulci using specific reference charts. A malformation of cortical development can be suspected because of the lack of visualization of a given structure, a reduced degree of folding of the fissure, or a smaller depth of the sulcus compared to what is expected for a given gestational age. In our daily practice, we usually measure Sylvian fissure length on a trans-thalamic plane tracing a vertical line perpendicular to the midline starting from the insula external border to the internal surface of the parietal bone; a parieto-occipital fissure is measured in a trans-ventricular plane at the point of maximum extension and symmetry compared to the contralateral fissure starting from the midline, avoiding the inclusion of cortical tissue in the measurement; a calcarine fissure was identified in a coronal view of the trans-cerebellar plane and measured by tracing a line from the apex of the fissure to the midline [22]. The findings from this systematic review showed that Sylvian and parieto-occipital fissures were visualized in all cases undergoing ultrasound assessment at the time of the anomaly scan, while calcarine fissures were detected in only half of the cases. However, only a few studies, including a very limited number of cases, reported cortical development in fetuses with no anomalies, thus potentially limiting the clinical applicability of these findings. Furthermore, these studies did not specifically evaluate the inter- and intra-observer variability in reporting the appearance of a given fissure and assessed the development of only of a limited number of brain sulci. Malformations of cortical development can present with an abnormal appearance of a limited area of the cortical surface of the brain in the presence of a normal appearance of the most reported brain sulci on prenatal ultrasound. The lack of a comprehensive and standardized approach to evaluating cortical anomalies in the fetus represents another peculiar issue. Most of the malformations of cortical development present in the setting of other subtle major CNS anomalies, such as ventriculomegaly or midline or posterior fossa anomalies. The identification of their presence is therefore crucial in all the CNS anomalies presented as isolated at the first ultrasound assessment and represents the major determinant of adverse neurological outcome in these fetuses [49,50]. However, while it is widely accepted that fetuses with isolated ventriculomegaly or isolated agenesis of the corpus callosum should undergo detailed neurosonography to identify cases at risk of abnormal cortical development, there is still no objective evidence on how to identify the presence of a cortical anomaly in a fetus not presenting with other CNS or extra-CNS malformations. Abnormal imaging appearance of the fetal head in axial view can suggest the presence of midline or posterior fossa anomalies, which can then be identified through specific diagnostic algorithms reported in the published literature. Conversely, a standardized approach to identifying cortical anomalies has not been fully reported yet. Some of these anomalies can be suspected when there is a reduced folding of a given fissure at ultrasound [32]. However, such assessment is subjective and potentially affected by a high rate of false positive diagnoses, leading to unnecessary parental anxiety. Objective evaluation of the growth of a given cortical structure should theoretically overcome this limitation. However, the present systematic review highlights the high risk of bias of most of the published charts regarding several crucial methodological aspects such as the study design or statistical analysis. In this scenario, charts for cortical development should be used with caution, especially in fetuses not presenting with other anomalies at neurosonography. In view of the limited evidence of normal cortical development and the lack of standardized imaging algorithms to diagnose malformations of cortical development, fetuses at high risk for these conditions should be referred to centers with high expertise in the prenatal diagnosis of these anomalies in order to provide parents a more accurate prognosis for their child. The findings from this systematic review also highlight the need for large studies specifically looking at the timing of the appearance of the different brain sulci and to construct robust reference charts to quantify their development. Standardized algorithms for prenatal assessment of fetuses at high risk of malformations of cortical development are also warranted in order to improve the ability of prenatal imaging to diagnose these anomalies.

## 5. Conclusions

There is still limited evidence from the published literature on the normal cortical development assessed on ultrasound, prenatal diagnosis of cortical anomalies, and associated malformations reported in the setting of such anomalies. More importantly, most of the published reference charts on cortical fissures show a high risk of bias either in the study design, statistical analysis, and reporting methods. The findings from this systematic review highlight the need for large studies specifically looking at the timing of the appearance of the different brain sulci and to construct robust reference charts to quantify their development. Standardized algorithms for prenatal assessment of fetuses at high risk of malformations of cortical development are also warranted in order to improve the ability of prenatal imaging to diagnose these anomalies.

## Figures and Tables

**Table 1:** General characteristics of the included studies.

Author	Ref	Year	Country	Study Design	Period Considered	Type of Cortical Anomalies	Fetuses (n)
Peero	[22]	2023	Israel	Retrospective cohort	NR	Prenatal diagnosis of malformations of cortical development	19
Cabet	[23]	2023	France	Retrospective cohort	NR	Prenatal diagnosis of malformations of cortical development	8
Marra	[24]	2023	Italy	Prospective, cross-sectional	2022	Sylvian, parieto-occipital, and calcarine fissures	344
Zeng	[25]	2023	China	Prospective, cross-sectional	2018–2020	Assessment of Sylvian fissure plateau angle	183
Yi	[26]	2023	China	Prospective, cross-sectional	2021	Assessment of Sylvian fissure through the measurement of the area and perimeter of the insula in 3D CVI imaging	286
Krajden Haratz	[27]	2022	Israel	Retrospective cohort	2012–2019	Prenatal diagnosis of malformations of cortical development	20
Chaithanya	[28]	2022	India	cross-sectional observational	NR	Evaluation of the timing of the appearance of cerebral sulci	241
Montaguti	[29]	2021	Italy	Retrospective cohort	NR	Prenatal diagnosis of malformations of cortical development	31
Rodriguez-Sibaja	[30]	2021	Multicenter	Longitudinal	2009–2016	Assessment of Sylvian fissure maturation	2359
Napolitano	[31]	2020	Multicenter	Prospective, cross-sectional	2009–2016	Sylvian and parieto-occipital fissures	442
Pooh	[32]	2019	Japan	Retrospective cohort	2010–2017	Prenatal diagnosis of malformations of cortical development	22
Spinelli	[33]	2018	Italy	Prospective, cross-sectional	NR	Sylvian fissure	329
Poon	[34]	2018	Japan	prospective, cross-sectional	March–December 2019	3D ultrasound assessment of Sylvian fissure	422
Chen	[35]	2017	China	Prospective, cross-sectional	2013	Sylvian, parieto-occipital, and calcarine fissures	746
Gindes	[36]	2015	Israel	Prospective, cross-sectional	2008–2009	Assessment of the perimeter and area of SF using 3D ultrasound	55
Contro	[37]	2015	Italy Italy	Prospective cohort	2012	Evaluation of the timing of the appearance of cerebral sulci	50
Alves	[38]	2013	Brazil	Prospective, cross-sectional	2010–2012	Sylvian, parieto-occipital, calcarine, and hyppocampal fissures	393
Alonso	[39]	2010	Spain	Prospective, cross-sectional	NR	Sylvian, parieto-occipital, and calcarine fissures	180
Pistorius	[40]	2010	Netherlands	Prospective, longitudinal	NR	Evaluation of the timing of the appearance of cerebral sulci	28
Guibaud	[41]	2008	France	Retrospective cohort	2001–2006	Prenatal diagnosis of malformations of cortical development	15
Quarello	[42]	2008	France	Prospective, cross-sectional	NR	Subjective assessment of Sylvian fissure	200
Malinger	[43]	2007	Israel	Prospective cohort	2000–2008	Prenatal diagnosis of malformations of cortical development	23
Mittal	[44]	2007	USA	Prospective, cross-sectional	2001–2005	Sylvian fissure	202
Cohen-Sacher	[45]	2006	Israel	Prospective, longitudinal	NR	Evaluation of the timing of the appearance of cerebral sulci	22
Correa	[46]	2006	Spain	Prospective	NR	Evaluation of the timing of the appearance of cerebral sulci	202
Fong	[47]	2004	Canada	Retrospective cohort	1999–2003	Prenatal diagnosis of malformations of cortical development	7
Toi	[48]	2004	Canada	Prospective	NR	Evaluation of the timing of the appearance of cerebral sulci	46

NR, not reported.

**Table 2:** Quality assessment of the included studies according to the Newcastle–Ottawa Scale (NOS) for cohort studies. A study can be awarded a maximum of one star for each numbered item within the Selection and Outcome categories. A maximum of two stars can be given for Comparability.

Author	Ref	Year	Selection	Comparability	Outcome
Peero	[22]	2023	★★	★★	★★
Cabet	[23]	2023	★★	★★	★★
Zeng	[25]	2023	★★	★★	★★
Yi	[26]	2023	★★	★	★★
Krajden Haratz	[27]	2022	★★	★	★
Chaithanya	[28]	2022	★★	★★	★★
Montaguti	[29]	2021	★★	★	★
Rodriguez-Sibaja	[30]	2021	★★	★★	★
Pooh	[32]	2019	★★	★	★★
Poon	[34]	2018	★★	★	★★
Gindes	[36]	2015	★★	★	★
Contro	[37]	2015	★★	★	★
Pistorius	[40]	2010	★★	★	★★
Guibaud	[41]	2008	★★	★	★★
Quarello	[42]	2008	★★	★★	★★
Malinger	[43]	2007	★★	★	★
Cohen-Sacher	[45]	2006	★★	★★	★★
Correa	[46]	2006	★★	★	★★
Fong	[47]	2004	★★	★★	★★
Toi	[48]	2004	★★	★	★

**Table 3:** Pooled proportions of the timing of the appearance of the Sylvian, parietal occipital, and calcarine fissures in the fetus according to different gestational ages.

Cortical Structure	Studies (n)	Visualized at 18–19 Weeks % (95% CI) [I^2^]	Visualized at 20–21 Weeks % (95% CI) [I^2^]	Visualized at 22–23 Weeks % (95% CI) [I^2^]	Visualized at >24 Weeks % (95% CI) [I^2^]
Sylvian fissure	6	97.69 (92.01–99.97) [74.8] 213/222 fetuses	98.17 (94.83–99.83) [53.1] 24/255 fetuses	98.94 (97.0–99.90) [0] 186/187 fetuses	100 (97.45–100) ^a^ [0] 164/164 fetuses
Parieto-occipital fissure	6	81.56 (48.41–99.26) ^a^ [74.8] 131/176 fetuses	96.59 (83.24–99.75) [92.8] 225/255 fetuses	96.85 (88.81–99.98) ^a^ [71.8] 136/141 fetuses	100 (96.85–100) [0] 118/118 fetuses
Calcarine fissure	5	37.27 (0.5–89.59) ^b^ [74.8] 59/126 fetuses	80.42 (50.20–98.23) [95.1] 155/204 fetuses	89.18 (73.97–98.19) [73.1] 82/91 fetuses	96.02 (84.67–100) ^c^ [65.6] 69/72 fetuses

^a^ Five studies are included in this meta-analysis. ^b^ Four studies are included in this meta-analysis. ^c^ Four studies are included in this meta-analysis.

**Table 4:** Pooled proportions for the association of the different anomalies in fetuses with MCD detected at ultrasound.

Outcome	Studies (n)	Fetuses (n/N)	Pooled Proportions % (95% CI)	I^2^ (%)
Associated CNS anomalies at ultrasound	9	123/130	93.79 (89.11–97.23)	0
Associated extra-CNS anomalies at ultrasound	8	49/125	38.84 (27.32–53.08)	55.9
Isolated findings at ultrasound	9	7/130	6.21 (2.77–10.89)	0
Ventriculomegaly	9	8/130	6.20 (0.69–16.67)	73.3
Midline anomalies	9	1/130	10.17 (1.80–24.24)	0
Posterior fossa anomalies	9	12/130	2.24 (0.43–5.41)	79
Multiple anomalies	9	117/130	82.63 (65.55–94.69)	80.6

CNS, central nervous system.

**Table 5:** Assessment of the published charts on fetal cortical structures by ultrasound (0 indicates a low risk of bias, while 1 indicates a high risk of bias).

Published Chart	Marra (2023)	Napolitano (2020)	Spinelli (2018)	Chen (2017)	Alves (2013)	Alonso (2010)	Mittal (2007)	Overall
Reference	[24]	[31]	[33]	[35]	[38]	[39]	[44]	
1.1. Study design	0	0	0	0	0	1	0	**1**
1.2 Sample selection	0	0	0	0	1	0	1	**2**
1.3 Number of scans per fetus	0	1	1	0	0	1	0	**3**
1.4. Inclusion/exclusion criteria	0	0	0	1	0	0	0	**1**
1.5 Sample size calculation	0	0	0	1	1	1	1	**4**
1.6 Data collection	0	0	0	0	0	0	0	**0**
1.7 Pregnancy dating	0	0	0	0	0	1	0	**1**
1.8 GA at inclusion calculation	0	0	1	0	1	1	1	**4**
2.1 Number of measurements taken for each biometric variable	1	0	1	0	1	1	0	**4**
2.2 statistical methods	0	0	0	0	0	0	0	**0**
2.3 assessment of increasing variability of data with gestation	0	0	1	1	1	1	0	**4**
2.4 assessment of goodness of fit of the model	0	0	0	1	1	1	1	**4**
2.5 scatterplot of data with fitted percentiles superimposed	0	0	0	1	0	0	0	**1**
2.6 Changes in reference percentiles across gestation	0	0	0	1	0	0	0	**1**
2.7 Methods used to estimate GA specific intervals for the measurement	0	0	1	1	1	1	0	**4**
3.1 Characteristics of study population	0	1	0	1	1	1	1	**5**
3.2 Description of number approached/enrolled	0	1	0	0	0	1	1	**3**
3.3 Ultrasound equipment used	0	0	0	0	0	0	0	**0**
3.4 Number of participating practitioners	0	0	0	0	0	0	0	**0**
3.5 Description of measurement technique(s)	0	0	0	0	0	0	0	**0**
3.6 Quality control measure	0	0	0	0	0	0	0	**0**
3.7 reporting of mean and SD of each measurement and sample size or each week of gestation	1	0	1	0	1	1	1	**5**
3.8 Reporting of regression equation for mean (and SD if relevant) for each measurement	1	0	0	0	0	1	1	**3**

Numbers in brackets refer to year of publication.

## Data Availability

The original contributions presented in this study are included in the article/Supplementary Materials. Further inquiries can be directed to the corresponding author.

## Supplementary Materials

The following supporting information can be downloaded at: https://www.mdpi.com/article/10.3390/biomedicines12071397/s1, Table S1: Excluded studies and reason for the exclusion.

## References

[1] Malinger G., Paladini D., Haratz K.K., Monteagudo A., Pilu G.L., Timor-Tritsch I.E. (2020). ISUOG Practice Guidelines (updated): Sonographic examination of the fetal central nervous system. Part 1: Performance of screening examination and indications for targeted neurosonography. Ultrasound Obstet. Gynecol..

[2] Paladini D., Malinger G., Birnbaum R., Monteagudo A., Pilu G., Salomon L.J., Timor-Tritsch I.E. (2021). ISUOG Practice Guidelines (updated): Sonographic examination of the fetal central nervous system. Part 2: Performance of targeted neurosonography. Ultrasound Obstet. Gynecol..

[3] Di Mascio D., Sileo F.G., Khalil A., Rizzo G., Persico N., Brunelli R., Giancotti A., Panici P.B., Acharya G., D’antonio F. (2019). Role of magnetic resonance imaging in fetuses with mild or moderate ventriculomegaly in the era of fetal neurosonography: Systematic review and meta-analysis. Ultrasound Obstet. Gynecol..

[4] ENSO Working Group (2020). Role of prenatal magnetic resonance imaging in fetuses with isolated mild or moderate ventriculomegaly in the era of neurosonography: International multicenter study. Ultrasound Obstet. Gynecol..

[5] Di Mascio D., Khalil A., Pilu G., Rizzo G., Caulo M., Liberati M., Giancotti A., Lees C., Volpe P., Buca D. (2021). Role of prenatal magnetic resonance imaging in fetuses with isolated severe ventriculomegaly at neurosonography: A multicenter study. Eur. J. Obstet. Gynecol. Reprod. Biol..

[6] Di Mascio D., Rizzo G., Khalil A., D’Antonio F., ENSO Working Group (2023). Role of fetal magnetic resonance imaging in fetuses with congenital cytomegalovirus infection: Multicenter study. Ultrasound Obstet. Gynecol..

[7] ENSO Working Group (2021). Role of prenatal magnetic resonance imaging in fetuses with isolated anomalies of corpus callosum: Multinational study. Ultrasound Obstet. Gynecol..

[8] Sileo F.G., Di Mascio D., Rizzo G., Caulo M., Manganaro L., Bertucci E., Masmejan S., Liberati M., D’amico A., Nappi L. (2021). Role of prenatal magnetic resonance imaging in fetuses with isolated agenesis of corpus callosum in the era of fetal neurosonography: A systematic review and meta-analysis. Acta Obstet. Gynecol. Scand..

[9] Di Mascio D., Buca D., Rizzo G., Khalil A., Timor-Tritsch I.E., Odibo A., Mappa I., Flacco M.E., Giancotti A., Liberati M. (2022). Methodological Quality of Fetal Brain Structure Charts for Screening Examination and Targeted Neurosonography: A Systematic Review. Fetal Diagn. Ther..

[10] Barkovich A.J., Kuzniecky R.I., Dobyns W.B., Jackson G.D., Becker L.E., Evrard P. (1996). A classification scheme for malformations of cortical development. Neuropediatrics.

[11] Barkovich A.J., Kuzniecky R.I., Jackson G.D., Guerrini R., Dobyns W.B. (2005). A developmental and genetic classification for malformations of cortical development. Neurology.

[12] De Robertis V., Sen C., Timor-Tritsch I., Chaoui R., Volpe P., Galindo A., Achiron R., Pooh R., Khalil A., Volpe N. (2021). WAPM-World Association of Perinatal Medicine Practice Guidelines: Fetal central nervous system examination. J. Perinat. Med..

[13] Henderson L.K., Craig J.C., Willis N.S., Tovey D., Webster A.C. (2010). How to write a Cochrane systematic review. Nephrology.

[14] NHS Centre for Reviews and Dissemination (2009). Systematic Reviews: CRD’s Guidance for Undertaking Reviews in Health Care.

[15] Page M.J., McKenzie J.E., Bossuyt P.M., Boutron I., Hoffmann T.C., Mulrow C.D., Shamseer L., Tetzlaff J.M., Akl E.A., Brennan S.E. (2021). The PRISMA 2020 statement: An updated guideline for reporting systematic reviews. BMJ.

[16] Ioannou C., Talbot K., Ohuma E., Sarris I., Villar J., Conde-Agudelo A., Papageorghiou A.T. (2012). Systematic review of methodology used in ultrasound studies aimed at creating charts of fetal size. BJOG.

[17] Corroenne R., Grevent D., Kasprian G., Stirnemann J., Ville Y., Mahallati H., Salomon L.J. (2023). Corpus callosal reference ranges: Systematic review of methodology of biometric chart construction and measurements obtained. Ultrasound Obstet. Gynecol..

[18] Newcastle-Ottawa Scale for Assessing the Quality of Non Randomised Studies in Meta-Analyses. http://www.ohri.ca/programs/clinical_epidemiology/oxford.asp.

[19] Stroup D.F., Berlin J.A., Morton S.C., Olkin I., Williamson G.D., Rennie D., Moher D., Becker B.J., Sipe T.A., Thacker S.B. (2000). Meta-analysis of observational studies in epidemiology: A proposal for reporting. Meta-analysis of Observational Studies in Epidemiology (MOOSE) group. JAMA.

[20] Hunter J.P., Saratzis A., Sutton A.J., Boucher R.H., Sayers R.D., Bown M.J. (2014). In meta-analyses of proportion studies, funnel plots were found to be an inaccurate method of assessing publication bias. J. Clin. Epidemiol..

[21] Manzoli L., De Vito C., Salanti G., D’Addario M., Villari P., Ioannidis J.P. (2011). Meta-analysis of the immunogenicity and tolerability of pandemic influenza A 2009 (H1N1) vaccines. PLoS ONE.

[22] Peero E.K., Kugelman N., Gindes L., Shariv A., Lev D., Tamarkin M., Haddad L., Bakry H., Weizman B., Shapiro I. (2023). Diagnosis of fetal cortical abnormalities by new reference charts for assessment of sylvian fissure biometry. Prenat. Diagn..

[23] Cabet S., Putoux A., Lesca G., Lesage A., Massoud M., Guibaud L., Collaborators (2024). Prenatal diagnosis of microcephaly with simplified gyral pattern: Series of 8 cases. Ultrasound Obstet. Gynecol..

[24] Marra M.C., Pietrolucci M.E., Mappa I., Lu J.L.A., Di Mascio D., D’Antonio F., Rizzo G. (2023). Modeling fetal cortical development by quantile regression for gestational age and head circumference: A prospective cross-sectional study. J. Perinat. Med..

[25] Zeng Q., Wen H., Liao Y., Luo D., Qin Y., Liang M., Li S. (2023). A New Parameter to Evaluate Fetal Sylvian Fissure by Transabdominal 2-D Ultrasound. Ultrasound Med. Biol..

[26] Yi F., Zhang C., Zou Y., Li X., Li J., Deng L., Chen L., Chen Z. (2023). Three-Dimensional Crystal Vue Imaging technology assessment of Sylvian fissures at 20-32+6 weeks’ normal gestation. Eur. Radiol..

[27] Krajden Haratz K., Birnbaum R., Kidron D., Har-Toov J., Salemnick Y., Brusilov M., Malinger G. (2023). Malformation of cortical development with abnormal cortex: Early ultrasound diagnosis between 14 and 24 weeks of gestation. Ultrasound Obstet. Gynecol..

[28] Chaithanya A., Sakalecha A.K., Srinivasa B.C.R. (2022). Role of Ultrasonography in the Evaluation of Normal Developmental Pattern of Fetal Cerebral Sulci Between 18 and 32 Weeks of Gestational Age. Cureus.

[29] Montaguti E., Bellussi F., Rizzo R., Livi A., Salsi G., Toni F., Maffei M., Lenzi J., Bonasoni M.P., Pilu G. (2021). Sylvian fossa sonographic measurements in 18 to 23 weeks fetuses with and without cerebral malformations. Am. J. Obstet. Gynecol. MFM.

[30] Rodriguez-Sibaja M.J., Villar J., Ohuma E.O., Napolitano R., Heyl S., Carvalho M., Jaffer Y.A., Noble J.A., Oberto M., Purwar M. (2021). Fetal cerebellar growth and Sylvian fissure maturation: International standards from Fetal Growth Longitudinal Study of INTERGROWTH-21stProject. Ultrasound Obstet. Gynecol..

[31] Napolitano R., Molloholli M., Donadono V., Ohuma E.O., Wanyonyi S.Z., Kemp B., Yaqub M.K., Ash S., Barros F.C., Carvalho M. (2020). International Fetal and Newborn Growth Consortium for the 21st Century (INTERGROWTH-21st). International standards for fetal brain structures based on serial ultrasound measurements from Fetal Growth Longitudinal Study of INTERGROWTH-21st Project. Ultrasound Obstet. Gynecol..

[32] Pooh R.K., Machida M., Nakamura T., Uenishi K., Chiyo H., Itoh K., Yoshimatsu J., Ueda H., Ogo K., Chaemsaithong P. (2019). Increased Sylvian fissure angle as early sonographic sign of malformation of cortical development. Ultrasound Obstet. Gynecol..

[33] Spinelli M., Sica C., Ghezzi F., Cromi A., Surbek D., Raio L. (2019). Nomograms of the Fetal Sylvian Fissure and Insular Lobe throughout Gestation: A Multicentric, Ultrasonographic Cross-Sectional Study. Fetal Diagn. Ther..

[34] Poon L.C., Sahota D.S., Chaemsaithong P., Nakamura T., Machida M., Naruse K., Wah Y.M., Leung T.Y., Pooh R.K. (2019). Transvaginal three-dimensional ultrasound assessment of Sylvian fissures at 18–30 weeks’ gestation. Ultrasound Obstet. Gynecol..

[35] Chen X., Li S.L., Luo G.Y., Norwitz E.R., Ouyang S.Y., Wen H.X., Yuan Y., Tian X.-X., He J.-M. (2017). Ultrasonographic Characteristics of Cortical Sulcus Development in the Human Fetus between 18 and 41 Weeks of Gestation. Chin. Med. J..

[36] Gindes L., Malach S., Weisz B., Achiron R., Leibovitz Z., Weissmann-Brenner A. (2015). Measuring the perimeter and area of the Sylvian fissure in fetal brain during normal pregnancies using 3-dimensional ultrasound. Prenat. Diagn..

[37] Contro E., Salsi G., Montaguti E., Morganelli G., Pilu G., Rizzo N., Bonasoni P., Ghi T. (2015). Sequential analysis of the normal fetal fissures with three-dimensional ultrasound: A longitudinal study. Prenat. Diagn..

[38] Alves C.M., Araujo Júnior E., Nardozza L.M., Goldman S.M., Martinez L.H., Martins W.P., Oliveira P.S., Moron A.F. (2013). Reference ranges for fetal brain fissure development on 3-dimensional sonography in the multiplanar mode. J. Ultrasound Med..

[39] Alonso I., Borenstein M., Grant G., Narbona I., Azumendi G. (2010). Depth of brain fissures in normal fetuses by prenatal ultrasound between 19 and 30 weeks of gestation. Ultrasound Obstet. Gynecol..

[40] Pistorius L.R., Stoutenbeek P., Groenendaal F., de Vries L., Manten G., Mulder E., Visser G. (2010). Grade and symmetry of normal fetal cortical development: A longitudinal two- and three-dimensional ultrasound study. Ultrasound Obstet. Gynecol..

[41] Guibaud L., Selleret L., Larroche J.C., Buenerd A., Alias F., Gaucherand P., Portes V.D., Pracros J. (2008). Abnormal Sylvian fissure on prenatal cerebral imaging: Significance and correlation with neuropathological and postnatal data. Ultrasound Obstet. Gynecol..

[42] Quarello E., Stirnemann J., Ville Y., Guibaud L. (2008). Assessment of fetal Sylvian fissure operculization between 22 and 32 weeks: A subjective approach. Ultrasound Obstet. Gynecol..

[43] Malinger G., Kidron D., Schreiber L., Ben-Sira L., Hoffmann C., Lev D., Lerman-Sagie T. (2007). Prenatal diagnosis of malformations of cortical development by dedicated neurosonography. Ultrasound Obstet. Gynecol..

[44] Mittal P., Goncalves L., Kusanovic J., Espinoza J., Lee W., Nien J.K., Soto E., Romero R. (2007). Objective evaluation of Sylvian fissure development by multiplanar 3-dimensional ultrasonography. J. Ultrasound Med..

[45] Cohen-Sacher B., Lerman-Sagie T., Lev D., Malinger G. (2006). Sonographic developmental milestones of the fetal cerebral cortex: A longitudinal study. Ultrasound Obstet. Gynecol..

[46] Correa F.F., Lara C., Bellver J., Remohí J., Pellicer A., Serra V. (2006). Examination of the fetal brain by transabdominal three-dimensional ultrasound: Potential for routine neurosonographic studies. Ultrasound Obstet. Gynecol..

[47] Fong K.W., Ghai S., Toi A., Blaser S., Winsor E.J., Chitayat D. (2004). Prenatal ultrasound findings of lissencephaly associated with Miller-Dieker syndrome and comparison with pre- and postnatal magnetic resonance imaging. Ultrasound Obstet. Gynecol..

[48] Toi A., Lister W.S., Fong K.W. (2004). How early are fetal cerebral sulci visible at prenatal ultrasound and what is the normal pattern of early fetal sulcal development?. Ultrasound Obstet. Gynecol..

[49] Pagani G., Thilaganathan B., Prefumo F. (2014). Neurodevelopmental outcome in isolated mild fetal ventriculomegaly: Systematic review and meta-analysis. Ultrasound Obstet. Gynecol..

[50] D’Antonio F., Pagani G., Familiari A., Khalil A., Sagies T.L., Malinger G., Leibovitz Z., Garel C., Moutard M.L., Pilu G. (2016). Outcomes Associated With Isolated Agenesis of the Corpus Callosum: A Meta-analysis. Pediatrics.

