# Supplementary material for: Sonographic Cortical Development and Anomalies in the Fetus: A Systematic Review and Meta-Analysis
Source: Biomedicines. 2024 Jun 24;12(7):1397. doi: 10.3390/biomedicines12071397 (PMC11273757; doi:10.3390/biomedicines12071397)
Supplement: Supplementary file 1 [file biomedicines-12-01397-s001.zip › biomedicines-3033264-supplementary.pdf]

Supplementary Table S1. Excluded studies and reason for the exclusion.

Author	Year	Title	Reason for the exclusion
Everwijin	2021	Serial neurosonography in fetuses with congenital heart defects shows mild delays in cortical development	No data for the outcomes observed in the present systematic review
Everwijin	2020	The association between flow and oxygenation and cortical development in fetuses with congenital heart defects using a brain-age prediction algorithm	No data for the outcomes observed in the present systematic review
Darouich	2019	Prenatal sonographic diagnosis of Dandy-Walker malformation and type III lissencephaly: A novel association	Case report
Everwijin	2019	Cortical development in fetuses with congenital heart defects using an automated brain-age prediction algorithm	No data for the outcomes observed in the present systematic review
Kappou	2016	Second-Trimester Sonographic Diagnosis of Polymicrogyria	case report
Lacalm	2016	Prenatal diagnosis of cobblestone lissencephaly associated with Walker–Warburg syndrome based on a specific sonographic pattern	case series
Teixeira	2015	Association of periventricular nodular heterotopia with posterior fossa cyst: a prenatal case series	Case series including 4 cases of PVNH
Businelli	2014	Ultrasound evaluation of cortical brain development in fetuses with intrauterine growth restriction	No data for the outcomes observed in the present systematic review
Cagneaux	2013	Pre- and postnatal imaging of early cerebral damage in Sturge-Weber syndrome	Case report
Li	2011	Ultrasound and MRI in fetuses with ventriculomegaly: Can cortical development be used to predict postnatal outcome?	No data for the outcomes observed in the present systematic review
Chen	2010	VENTRICULOMEGALY, INTRAUTERINE GROWTH RESTRICTION, AND CONGENITAL HEART DEFECTS AS SALIENT PRENATAL SONOGRAPHIC FINDINGS OF MILLER-DIEKER LISSENCEPHALY SYNDROME ASSOCIATED WITH MONOSOMY 17P (17P13.2 □ PTER) IN A FETUS	Case report
Aslan	2009	Prenatal Diagnosis of Lissencephaly: A Case Report	Case report
Lin	2009	Prenatal diagnosis of monosomy 17p (17p13.3-->pter) associated with polyhydramnios, intrauterine growth restriction, ventriculomegaly, and Miller-Dieker lissencephaly syndrome in a fetus.	case report
Dhombres	2008	Prenatal ultrasonographic diagnosis of polymicrogyria	Case report
Fuchs	2008	Prenatal and Postnatal Follow-Up of a Fetal Interhemispheric Arachnoid Cyst with Partial Corpus Callosum Agenesis, Asymmetric Ventriculomegaly and Localized Polymicrogyria	Case report
Saltzman	1991	Prenatal diagnosis of Lissencephaly	Case Report
Saltzman	1991	Prenatal diagnosis of lissencephaly	Case report
